# Predictors of Complete Response and Disease Recurrence Following Chemoradiation for Rectal Cancer

**DOI:** 10.3389/fonc.2015.00286

**Published:** 2015-12-22

**Authors:** Danielle S. Bitterman, Lucas Resende Salgado, Harvey G. Moore, Nicholas J. Sanfilippo, Ping Gu, Ioannis Hatzaras, Kevin L. Du

**Affiliations:** ^1^Department of Radiation Oncology, New York University Langone Medical Center, New York, NY, USA; ^2^Division of Colon and Rectal Surgery, New York University Langone Medical Center, New York, NY, USA; ^3^Division of Hematology and Oncology, New York University Langone Medical Center, New York, NY, USA; ^4^Division of Surgical Oncology, New York University Langone Medical Center, New York, NY, USA

**Keywords:** rectal cancer, radiotherapy, clinical predictors, complete response, watch-and-wait strategy

## Abstract

**Objective:**

Approximately 10–40% of rectal patients have a complete response (CR) to neoadjuvant chemoradiation (CRT), and these patients have improved survival. Thus, non-operative management (“watch-and-wait” approach) may be an option for select patients. We aimed to identify clinical predictors of CR following CRT.

**Methods:**

Patients treated with definitive CRT for T3–T4, locally unresectable T1–T2, low-lying T2, and/or node-positive rectal cancer from August 2004 to February 2015 were retrospectively reviewed. Most patients were treated with 50.4 Gy radiation and concurrent 5-fluoruracil or capecitabine. Patients were considered to have a CR if surgical pathology revealed ypT0N0M0 (operative management), or if they had no evidence of residual disease on clinical and radiographic assessment (non-operative management). Statistical analysis was carried out to determine predictors of CR and long-term outcomes.

**Results:**

Complete records were available on 138 patients. The median follow-up was 24.5 months. Thirty-six patients (26.3%) achieved a CR; 30/123 operatively managed patients (24.5%) and 6/15 (40%) non-operatively managed patients. None of the 10 patients with mucinous adenocarcinoma achieved a CR. Carcinoembryonic antigen (CEA) ≥5 μg/L at diagnosis (OR 0.190, 95% CI 0.037–0.971, *p* = 0.046), tumor size ≥3 cm (OR 0.123, 95% CI 0.020–0.745, *p* = 0.023), distance of tumor from the anal verge ≥3 cm (OR 0.091, 95% CI 0.013–0.613, *p* = 0.014), clinically node-positive disease at diagnosis (OR 0.201, 95% CI 0.045–0.895, *p* = 0.035), and interval from CRT to surgery ≥8 weeks (OR 5.267, 95% CI 1.068–25.961, *p* = 0.041) were independent predictors of CR. The CR group had longer 3-year distant metastasis-free survival (DMFS) (93.7 vs. 63.7%, *p* = 0.016) and 3-year disease-free survival (DFS) (91.1 vs. 67.8%, *p* = 0.038). Three-year locoregional control (LRC) (96.6 vs. 81.3%, *p* = 0.103) and overall survival (97.2 vs. 87.5%, *p* = 0.125) were higher in the CR group but this did not achieve statistical significance. CR was not an independent predictor of LRC, DMFS, or DFS.

**Conclusion:**

CEA at diagnosis, tumor size, tumor distance from the anal verge, node positivity at diagnosis, and interval from CRT to surgery were predictors of CR. These clinical variables may offer insight into patient selection and timing of treatment response evaluation in the watch-and-wait approach.

## Introduction

The standard treatment of locally advanced rectal cancer consists of chemoradiation (CRT), followed by total mesorectal excision (TME) 4–8 weeks later. Interestingly, in the German CAO/ARO/AIO-94 randomized phase III trial that established the superiority of preoperative CRT over postoperative CRT, 9% of patients achieved a pathologic complete response (pCR), defined as ypT0N0M0, following CRT. Other studies have reported a pCR rates ranging from ~10 to 40% (1–5). Further, Maas et al. demonstrated that achievement of a pCR is associated with an improved outcome (2).

These findings raised interest in the possibility of a non-operative treatment strategy, the “watch-and-wait” approach, for rectal cancer. Significant morbidity is associated with TME, including postoperative complications, creation of a definitive stoma, urinary and fecal incontinence, and sexual dysfunction. With the watch-and-wait approach, patients achieving a clinical complete response (cCR) have no immediate surgery, but instead are followed closely clinically and radiographically (6). Recent studies have reported rectal preservation rates of up to 78% and similar survival to patients treated operatively (7–10).

However, challenges remain in identifying which patients would benefit most from the watch-and-wait approach. Neither digital rectal examination (DRE), nor endoscopy, nor imaging is a perfect predictor of pCR (11–15). Thus, more accurate tests are needed in order to offer non-operative management to all patients who may benefit. Several studies have examined predictors of complete response (CR); however, few have assessed how radiotherapy (RT) treatment parameters affect response to CRT and most do not correlate predictors with long-term outcomes. Thus, the aim of this study was to determine clinical or pathologic factors that are prognostic of achievement of a CR as well as long-term oncologic outcomes following CRT for rectal cancer.

## Materials and Methods

### Study Sites and Patient Inclusion Criteria

We conducted a chart review on all patients who had received CRT for rectal adenocarcinoma at the NYU Department of Radiation Oncology between August 2004 and February 2015. Patients with T3–T4, locally unresectable T1–T2, low-lying T2, and/or node-positive rectal cancer were included in this study. Patients were excluded if they had stage IV disease at the start of treatment, if they were referred for palliative RT, or if pathology was not consistent with adenocarcinoma. Ten patients were excluded due to insufficient treatment data. We performed a retrospective analysis of the remaining 138 patients.

This study was approved by the New York University School of Medicine Institutional Review Board. Consent was waived as it was deemed that there was no more than minimal risk to research participants, the rights and welfare of the participants were not adversely affected, and the research could not be practicably conducted without a waiver.

### Diagnosis and Treatment

Patients were clinically staged with colonoscopy with biopsy, endoscopic ultrasonography ± pelvic MRI, and chest/abdomen/pelvis CT. External beam RT was delivered as either three-dimensional RT or intensity modulated RT with continuous standard fractionation. The pelvis was treated to a dose of 45 Gy, and gross disease received an additional dose of 5.4 Gy. All patients were treated in 1.8 Gy fractions. Most patients were treated concurrently with either continuous infusion 5-fluoruracil (5-FU) 225 mg/m^2^ over 24 h for 7 days/week or capecitabine 825 mg/m^2^ twice a day for 5 days/week. A minority of patient received an oxaliplatin-containing regimen with either 5-FU or capecitabine.

Prior to February 2014, patients underwent either low anterior resection (LAR) or abdominoperineal resection (APR) 5–12 weeks following neoadjuvant CRT. Patients who were not operative candidates or who preferred to not undergo surgery were followed per institutional routine.

After February 2014, patients with tumors assessable by DRE were managed per a formal watch-and-wait clinical protocol. Tumor response was assessed 10 weeks from the completion of RT with physical exam and DRE performed by the surgeon, flexible sigmoidoscopy, MRI ± PET/CT, and carcinoembryonic antigen (CEA) level. A cCR was defined as absence of residual tumor, ulceration, or rectal wall irregularity on both clinical and radiologic assessment. Radiologic features of cCR included presence of residual low-signal intensity and absence of restriction to diffusion on MRI, or absence of residual FDG avidity in the rectal wall on PET/CT. Determination of cCR was made by a multidisciplinary tumor board, which reviewed the above clinical factors and imaging. Patients who did not achieve a cCR underwent LAR or APR 5–12 weeks following neoadjuvant CRT. Patients achieving a cCR were followed with physical exam and DRE, CEA level, and flexible sigmoidoscopy every 2 months in year 1, every 3 months in year 2, every 6 months in years 3–5, and yearly thereafter. Radiologic assessment consisted of chest/abdomen/pelvic CT and MRI ± PET/CT every 6 months in years 1–2 and yearly thereafter. Patients who developed a local recurrence were offered standard TME surgery.

One hundred and twenty-two patients were treated prior to February 2014 and 16 patients were treated after. For all patients undergoing operative management, pCR was defined as ypT0N0M0.

### Data

Data were collected on patient age at diagnosis, gender, histology, clinical stage at presentation, neutrophil and lymphocyte count at diagnosis and after CRT, and CEA level at diagnosis. Date of pathologic diagnosis, dates of RT, RT dose, presence of unplanned RT breaks, chemotherapy regimens, surgery date, surgical procedure, and pathologic findings at surgery were recorded.

### Statistical Analysis

Outcome measures were achievement of either pCR (operative management) or cCR (non-operative management) (“CR”), locoregional control (LRC), distant metastasis-free survival (DMFS), disease-free survival (DFS), and overall survival (OS). LRC was defined as the interval from the end of RT to local or regional failure or most recent follow-up. DMFS was defined as the interval from the end of RT to distant failure or most recent follow-up. DFS was defined as the interval from the end of RT to the occurrence of local, regional, or distant failure, death, or most recent follow-up. OS was defined as the interval from the end of RT to the time of death by any cause or most recent follow-up.

Clinical and pathologic variables were compared between the CR and no CR groups using Student’s *t*-test to compare means and χ^2^ test to compare frequency. Univariable and multivariable binary logistic regression was used to assess predictors of CR. Survival curves for LRC, DMFS, DFS, and OS were created using the Kaplan–Meier method and compared with the log-rank test. Univariable and multivariable Cox proportional hazards regression was used to assess for predictors of LRC, DMFS, and DFS. CEA ≥5 μg/L at diagnosis was used as a cut-off as this is considered the normal value for smokers. Cut-off values for other continuous data were determined by the point on the receiver operator curve that maximized sensitivity and specificity. Patients who did not achieve a cCR and did not undergo surgery were excluded from the survival analysis. For all regression analyses, variables that predicted the dependent variable with a *p* value <0.200 on univariable analysis were considered in the multivariable analysis. We controlled for missing CEA and chemotherapy data in the regression models when applicable and found they were not significant except where noted.

All statistical tests were two-sided and *p* values <0.05 were considered significant. Statistical analyses were carried out using SPSS version 20 (IBM Corp., Armonk, NY, USA).

## Results

In our cohort, 36 patients achieved a CR (26.3%). One hundred and twenty-three patients underwent surgery following CRT, and 15 patients were managed non-operatively. Of the patients who were managed operatively, 30 (24.5%) achieved a pCR.

Of the patients managed non-operatively, four were found to have metastatic disease immediately following completion of CRT. Four patients did not achieve a cCR, but did not undergo surgery due to poor performance status or patient preference, and were excluded from the survival analysis. The remaining six patients (40%) achieved a cCR, the median time to cCR among these patients was 80 days [interquartile range (IQR) 40.3–261.5 days], and the median follow-up was 21.5 months (IQR 5.5–50.8 months).

### Patient Population and Characteristics

Demographic and clinical variables of the patients are reported in Table 1. The mean age of patients at diagnosis overall was 57.9 ± 13.4 years with no significant difference between patients who achieved a CR and those who did not (*p* = 0.445). The 37.7% of patients were female, and the gender distribution was similar between patient achieving a CR and those who did not (*p* = 0.531). Mean distance of the tumor from the anal verge was greater in the no CR group compared to the CR group (6.3 ± 3.5 vs. 4.5 ± 3.5 cm, *p* = 0.020). Mean CEA at diagnosis was significantly lower in patients achieving a CR than those who did not (3.1 ± 2.5 vs. 24.3 ± 80.5 μg/L, *p* = 0.024). CEA data were missing on 31 patients (22.5%); there was no difference in the proportion of missing data in the CR and no CR groups (*p* = 0.332).

**Table 1:** **Baseline patient demographics and clinical characteristics**.

Variables	Total (*n* = l38)	CR (*n* = 36)	No CR (*n* = 102)	*p* Value
Mean age ± SD (year)	57.9 ± 13.4	59.3 ± 12.3	57.4 ± 13.8	0.445
Female (%)	52 (37.7%)	12 (33.3%)	40 (39.2%)	0.531
Mean distance from anal verge ± SD (cm)	5.9 ± 3.6	4.5 ± 3.6	6.3 ± 3.5	**0.020**
Mean tumor size ± SD (cm)	5.4 ± 3.0	5.0 ± 3.9	5.6 ± 2.7	0.535
Differentiation (%)				0.989
Well	8 (5.8%)	2 (5.6%)	6 (5.9%)	
Moderate	85 (61.6%)	20 (55.6%)	65 (63.7%)	
Poor	16 (11.6%)	4 (11.1%)	12 (11.8%)	
Unknown	29 (21.0%)	10 (27.8%)	19 (18.6%)	
Histology (%)				0.121
Adenocarcinoma	127 (92.0%)	36 (100%)	91 (89.2%)	
Mucinous adenocarcinoma	10 (7.2%)	0	10 (9.8%)	
Adenocarcinoma with neuroendocrine features	1 (0.7%)	0	1 (1.0%)	
cT (%)				0.224
1	3 (2.2%)	2 (5.6%)	1 (1.0%)	
2	16 (11.6%)	5 (13.9%)	11 (10.8%)	
3	108 (78.3%)	28 (77.8%)	80 (78.4%)	
4	8 (5.8%)	0	8 (7.8%)	
*x*	3 (2.2%)	1 (2.8%)	2 (2.0%)	
cN (%)				0.135
0	44 (31.9%)	17 (47.2%)	27 (26.5%)	
1	71 (51.4%)	15 (41.7%)	56 (54.9%)	
2	22 (15.9%)	4 (11.1%)	18 (17.6%)	
*x*	1 (0.7%)	0	1 (1.0%)	
CEA				
Mean CEA ± SD (μg/L)	18.4 ± 68.9	3.l ± 2.5	24.3 ± 80.5	**0.024**
CEA ≥ 5 μg/L (%)	45 (32.6%)	6 (16.7%)	39 (38.2%)	**0.004**
Missing data	31 (22.5%)	6 (16.7%)	25 (24.5%)	0.332
Mean neutrophil count ± SD (cells/μL)	4.5 ± l.7	4.9 ± 2.3	4.4 ± l.5	0.252
Mean lymphocyte count ± SD (cells/μL)	1.8 ± 0.6	1.8 × 0.6	1.8 ± 0.7	0.993
Median NLR ± SD	3.0 ± 2.2	3.3 ± 3.2	2.9 ± l.7	0.389
NLR ≥5 at diagnosis (%)	13 (9.4%)	2 (5.6%)	11 (10.8%)	0.433

*SD, standard deviation; CEA, carcinoembryonic antigen; NLR, neutrophil:lymphocyte ratio*.

*The p Values that are significant (p<0.05) are bolded*.

### Treatment Characteristics

Chemoradiation characteristics are presented in Table 2. Four patients did not receive concurrent chemotherapy. Patients achieving a CR received a lower RT dose to the pelvis (4337 ± 559 vs. 4473 ± 173 cGy, *p* = 0.030). The two groups were otherwise homogeneous in terms of their CRT regimens and received induction and consolidation (post-CRT) chemotherapy in similar proportions.

**Table 2:** **Characteristics of chemoradiation**.

Variables	Total (*n* = 138)	CR (*n* = 36)	No CR (*n* = 102)	*p* Value
Mean time to CRT ± SD (days)	61.8 ± 82.3	53.2 ± 74.1	61.3 ± 85.3	0.903
Mean RT dose ± SD (cGy)	4994 ± 334	4942 ± 502	5012 ± 254	0.287
Mean RT dose to pelvis ± SD (cGy)	4439 ± 322	4337 ± 559	4473 ± 173	**0.030**
Mean boost to gross disease ± SD (cGy)	532 ± 211	530 ± 247	534 ± 199	0.931
Mean RT duration ± SD (days)	40.2 ± 6.2	39.0 ± 5.6	40.6 ± 6.3	0.171
RT interruption (%)	31 (22.5%)	7 (19.4%)	24 (23.5%)	0.614
Concurrent chemotherapy (%)				0.382
5-FU or capecitabine	119 (86.2%)	33 (91.7%)	86 (90.5%)	
Oxaliplatin-containing regimen^a^	8 (5.8%)	3 (8.3%)	5 (5.3%)	
None	4 (2.9%)	0	4 (4.2%)	
Missing data	7 (5.1%)	0	7 (6.7%)	
Additional chemotherapy (%)				0.593
Induction	7 (5.1%)	1 (2.8%)	6 (5.9%)	
Consolidation	8 (5.8%)	3 (8.3%)	5 (4.9%)	

*SD, standard deviation; CRT, chemoradiation; RT, radiation therapy; SD, standard deviation; 5-FU, 5 fluorouracil*.

*^a^Chemotherapy regimen containing oxaliplatin plus 5-FU or capecitabine*. *The p Values that are significant (p<0.05) are bolded*.

Operative management was similar in the CR and no CR groups; however, significantly more patients underwent APR in the CR group compared to the no CR group (36.7 vs. 15.1%, *p* = 0.016) (Table 3). One patient with a low-lying T2N0M0 rectal cancer underwent transanal excision and pathology revealed pT0Nx.

**Table 3:** **Characteristics of surgical management**.

Variables	Total (*n* = 123)	CR (*n* = 30)	No CR (*n* = 93)	*p* Value
CRT to surgery interval ≥8 weeks (%)	67 (54.4%)	21 (70.0%)	46 (49.5%)	0.059
Surgical procedure (%)				**0.016**
LAR	96 (78.0%)	18 (60%)	78 (83.9%)	
APR	25 (20.3%)	11 (36.7%)	14 (15.1%)	
Transanal excision	1 (0.8%)	1 (3.3%)	0	
Proctectomy	1 (0.8%)	0	1 (1.1%)	
Mean LNs resected ± SD (*n*)	17.9 ± 10.4	14.5 ± 7.4	19.0 ± 11.0	**0.013**
ypT (%)				<0.001
0	32 (26.0%)	30 (100%)	2 (2.2%)	
1	6 (4.9%)	–	6 (6.5%)	
2	28 (22.8%)	–	28 (30.1%)	
3	52 (42.3%)	–	52 (55.9%)	
4	3 (2.4%)	–	3 (3.2%)	
*x*	2 (1.6%)	–	2 (2.2%)	
ypN (%)				<0.001
0	78 (63.4%)	30 (100%)	48 (51.6%)	
1	28 (22.8%)	–	28 (30.1%)	
2	16 (13.0%)	–	16 (17.2%)	
*x*	1 (0.8%)	–	1 (1.1%)	
ypM^+^ (%)	4 (3.3%)	–	4 (4.3%)	0.197
Positive margins (%)	6 (4.9%)	–	6 (6.5%)	0.154
LVI (%)	25 (20.3%)	–	25 (26.9%)	0.01

*SD, standard deviation; CRT, chemoradiation; LAR, low anterior resection; APR, abdominoperineal resection; LN, lymph node; LVI lymphovascular invasion*.

*The p Values that are significant (p<0.05) are bolded*.

### Predictors of Complete Response

There was no difference in patient age (*p* = 0.465), gender (*p* = 0.691), distance of the tumor from the anal verge (*p* = 0.515), tumor size (*p* = 0.473), differentiation (*p* = 0.395), cT stage (*p* = 0.582), cN stage (*p* = 0.628), CEA level (*p* = 0.401), or neutrophil to lymphocyte ratio (NLR) level (*p* = 0.650) between pCR and cCR patients. pCR and cCR patients received similar RT doses (*p* = 0.485 for pelvic dose and *p* = 0.293 for boost dose), and concurrent chemotherapy (*p* = 0.089). While none of the operatively managed patients received induction or consolidation chemotherapy, one cCR patient received induction chemotherapy and three cCR patients received consolidation chemotherapy (*p* < 0.001). None of the 10 patients with mucinous adenocarcinoma achieved a CR.

On univariable analysis, tumor ≥3 cm from the anal verge, tumor size ≥3 cm, CEA ≥5 μg/L at diagnosis, clinically node-positive disease at diagnosis, increased RT duration, RT dose to the pelvis, and interval from CRT to surgery <8 weeks predicted against CR. On multivariable analysis, CEA ≥5 μg/L at diagnosis (OR 0.190, 95% CI 0.037–0.971, *p* = 0.046), tumor size ≥3 cm (OR 0.123, 95% CI 0.020–0.745, *p* = 0.023), distance of tumor from the anal verge ≥3 cm (OR 0.091, 95% CI 0.013–0.613, *p* = 0.014), clinically node-positive disease at diagnosis (OR 0.201, 95% CI 0.045–0.895, *p* = 0.035), and interval from CRT to surgery ≥8 weeks (OR 5.267, 95% CI 1.068–25.961, *p* = 0.041) were independent predictors of CR (Table 4).

**Table 4:** **Predictors of complete response to chemoradiation**.

	Univariable regression			Multivariable regression		
Covariables	OR	95% CI	*p* value	OR	95% CI	*p* Value
≥3 cm from anal verge	0.362	0.142–0.925	0.034	0.091	0.013–0.613	**0.014**
Tumor size ≥3 cm	0.269	0.079–0.918	0.036	0.123	0.020–0.745	**0.023**
CEA ≥5 μg/L at diagnosis	0.244	0.90–0.662	0.006	0.190	0.037–0.971	**0.046**
cN^+^	0.408	0.185–0.898	0.026	0.201	0.045–0.895	**0.035**
RT duration (days)	0.953	0.887–1.025	0.197	–	–	NS
RT dose to pelvis (cGy)	0.999	0.998–1.000	0.078	–	–	NS
CRT to surgery interval ≥8 weeks	2.384	0.989–5.759	0.053	5.267	1.068–25.961	**0.041**

*OR, odds ratio; CI, confidence interval; CEA, carcinoembryonic antigen; RT, radiation therapy; CRT, chemoradiation*.

*The p Values that are significant (p<0.05) are bolded*.

### Oncologic Outcomes

The median follow-up time was 24.5 months (IQR 10.0–49.3 months), and this was similar in the CR and no CR group (*p* = 0.936). At the time of this review, 123 patients were alive and 15 patients were deceased. Seven patients were lost to clinical follow-up by 1 year following treatment. In our population overall, mean OS was 106.8 months (95% CI 77.210–116.447 months) and 3-year OS was 90.0%.

**Figure 1:**
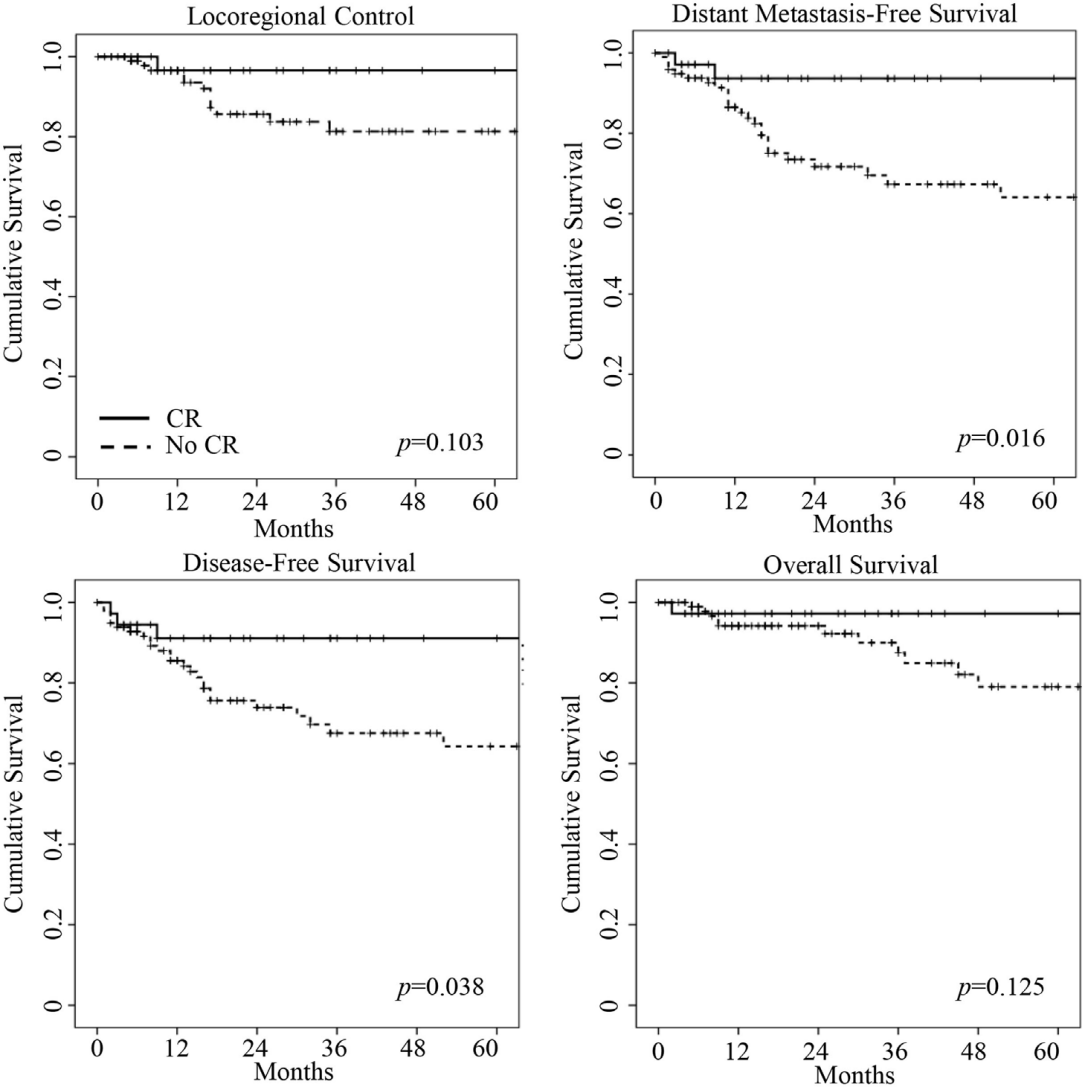
**Kaplan–Meier curves of locoregional control, distant metastasis-free survival, disease-free survival, and overall survival for complete response (CR) patients (bold line) and no complete response (no CR) patients (dashed line)**. CR patients had longer distant metastasis-free survival and disease-free survival on log-rank test (*p* values shown in graphs).

In the pCR group, one patient failed locoregionally, and one patient failed both locoregionally and distantly. None of the cCR patients have recurred. Kaplan–Meier curves for LRC, DMFS, DFS, and OS are presented in Figure 1. Three-year DMFS (93.7 vs. 63.7%, *p* = 0.016) and 3-year DFS (91.1 vs. 67.8%, *p* = 0.038) were longer among patients with a CR. While the CR group had longer 3-year LRC (96.6 vs. 81.3%, *p* = 0.103) and 3-year OS (97.2 vs. 87.5%, *p* = 0.125), this did not reach statistical significance.

On multivariable analysis, concurrent chemotherapy (*p* = 0.022), metastatic disease at surgery (ypM^+^) (0.028), and number of lymph nodes resected (*p* = 0.001) were predictive of locoregional failure. Increased absolute lymphocyte count (*p* = 0.007), concurrent chemotherapy (*p* < 0.001), and ypM^+^ (*p* < 0.001) were predictive of distant metastasis. Consolidation chemotherapy was associated with increased chance of distant metastasis on univariable analysis (*p* < 0.001), but could not be included on multivariate analysis due to collinearity with ypM^+^. When consolidation chemotherapy was included in the multivariable model instead of ypM^+^, it was a significant predictor of distant metastasis (HR 5.330, 95% CI 1.524–18.639, *p* = 0.009). Male gender (*p* = 0.020), mucinous adenocarcinoma histology (*p* = 0.010), CEA ≥5 μg/L at diagnosis (*p* = 0.039), and presence of an unplanned treatment break (*p* = 0.043) were associated with disease recurrence (Table 5). When consolidation chemotherapy was considered in the multivariable model instead of ypM^+^, it was not a significant predictor of disease recurrence. Importantly, missing concurrent chemotherapy data were significant in the multivariable models for locoregional recurrence (*p* < 0.001) and distant recurrence (*p* = 0.003) but not for disease recurrence.

**Table 5:** **Univariable and multivariable analysis of long-term oncologic outcomes**.

Covariables	Univariable regression			Multivariable regression		
	HR	95% CI	*p* value	HR	95% CI	*p* Value
**Locoregional failure**						
cN^+^	38.153	0.302–4824.220	0.140	–	–	NS
Neutrophil count at diagnosis	1.244	0.929–1.666	0.142	–	–	NS
Treatment break	3.286	1.102–9.800	0.033	–	–	NS
Concurrent chemotherapy	0.128	0.016–1.018	0.052	0.081	0.009–0.698	**0.022**
ypM^+^	6.735	1.486–30.528	0.013	12.212	1.314–113.477	**0.028**
LNs resected	1.045	1.007–1.085	0.021	1.066	1.026–1.107	**0.001**
CR following CRT	0.215	0.028–1.655	0.140	–	–	NS
**Distant failure**						
Lymphocyte count at diagnosis (cells/μL)	0.506	0.262–0.978	0.043	0.275	0.108–0.700	**0.007**
CEA ≥5 μg/L at diagnosis	2.846	1.101–7.357	0.031	–	–	NS
Treatment break	0.2619	1.212–5.662	0.014	–	–	NS
Total RT dose (cGy)^a^	1.001	0.999–1.004	0.168			
RT boost to gross disease (cGy)	1.001	1.000–1.003	0.068	–	–	NS
Concurrent chemotherapy	0.114	0.034–0.384	<0.001	0.025	0.004–0.153	<**0.001**
Induction chemotherapy	2.794	0.648–12.043	0.168	–	–	NS
Consolidation chemotherapy^b^	3.750	1.292–10.888	<0.015			
ypM^+^	9.531	3.206–28.332	<0.001	21.836	5.078–93.896	<**0.001**
**Disease recurrence**						
Male gender	0.427	0.200–0.028	0.028	0.337	0.135–0.840	**0.020**
Mucinous adenocarcinoma	3.382	1.282–8.921	0.014	4.477	1.436–13.951	**0.010**
CEA ≥5 μg/L at diagnosis	2.185	0.904–5.283	0.083	2.675	1.050–6.818	**0.039**
Treatment break	2.408	1.125–5.155	0.024	2.696	1.030–7.057	**0.043**
Total RT dose^a^ (cGy)	1.001	0.999–1.003	0.172			
RT boost to gross disease (cGy)	1.001	1.000–1.003	0.070	–	–	NS
Concurrent chemotherapy	0.169	0.040–0.716	0.016	–	–	NS
Consolidation chemotherapy^b^	2.770	0.832–9.215	0.097			
ypN^+^	2.217	0.992–4.851	0.052	–	–	NS
ypM^+^	6.031	1.781–20.420	0.004	–	–	NS
CR following CRT	0.304	0.092–1.009	0.052	–	–	NS

*HR, hazard ratio; CI, confidence interval; RL, reference level; CR, complete response; CRT, chemoradiation; RT, radialion therapy; CEA, carcinoembryonic antigen; LN, lymph node*.

*^a^Not included in multivariable analysis due to collinearity with RT to gross disease*.

*^b^Not included in multivariable model for distant recurrence due to collinearity with ypM^+^*. *The p Values that are significant (p<0.05) are bolded*.

## Discussion

Several studies have assessed the ability of clinical and radiographic data to accurately detect a cCR; however, there is evidence that these strategies miss many complete responders (11–15). In fact, a recent study found that 74% of patients with a pCR had a residual mucosal abnormality on gross examination (16). Given this, clinical and pathologic variables that are associated with a CR will likely continue to be valuable in risk-stratifying rectal cancer patients for the watch-and-wait strategy. We found CEA <5 μg/L at diagnosis, tumor size <3 cm, tumor distance <3 cm from the anal verge, clinically node-negative disease, and interval from the end of CRT to surgery ≥8 weeks to be predictive of CR following CRT for rectal cancer. Patients who achieved a CR had significantly longer DMFS and DFS. In addition, LRC and OS were longer among CR patients but this did not achieve statistical significance, likely due to the small sample size. While OS in the entire population was similar to nationally reported averages, OS among CR patients was much higher (17, 18). CR was not an independent predictor of LRC, DMFS, or DFS.

Few studies have assessed clinical or pathologic predictors of cCR. In their prospective trials of the watch-and-wait approach, Habr-Gama et al. have found no clinical differences between complete clinical responders and non-complete responders (19) and no independent predictors of cCR (20). Several other small, single-institutional studies have aimed to identify predictors of pCR. As evidence suggests that cCR and pCR outcomes are equivalent, we believe that combining pCR and cCR data to assess for predictors of CR is a valid strategy to identify potential clinical predictors (7, 9, 21). Further, the similar distribution of clinical characteristics between the cCR and pCR groups in our cohort supports the validity of combining the two populations. We hope that our experience will contribute to our ability to accurately predict CR.

Morphologic characteristics of the tumor, including size, mobility, circumferentiality, ulceration, and distance of the tumor from the anal verge have all been identified as predictors of pCR (22–27). In our cohort, both increased tumor size and increased distance from the anal verge predicted against CR. As two other studies have also found decreasing tumor size to predict for CR, it is reasonable to consider this variable when risk-stratifying patients for the watch-and-wait approach (26, 27). Interestingly, Restivo et al. found tumors >5 cm from the anal verge to be predictive of pCR (25). This conflicts with our own findings that more distal tumors predict CR. In our cohort, this association remained when non-operatively managed patients were excluded from the analysis (data not shown). While we reported similar median distance from the anal verge as Restivo et al., our IQR was wider and thus it is possible that very distal tumors are more susceptible to CRT. As distal rectal cancers are more amenable to clinical surveillance, more data are needed to understand whether distance from the anal verge is predictive of CRT response.

Carcinoembryonic antigen, currently used to monitor for disease recurrence in colorectal cancer, has been investigated as a biomarker for response to CRT. In agreement with our findings, many studies have found CEA level at the time of diagnosis to be associated with CR (24, 25, 27–32). Although 22.5% of CEA levels were missing, this was not significant when controlled for on regression analysis and our consistency with other studies suggests this relationship is real. While we did not evaluate post-CRT CEA levels, this has also been found to be predictive of pCR in other studies (27, 30, 33). The reproducibility of these findings among so many populations suggests that this variable is a reliable predictor of sensitivity to CRT.

In agreement with our findings, clinical node positivity has been shown by two other retrospective studies to be independently associated with decreased chance of achieving a CR (26, 32). Given these findings, it is possible that clinical node positivity may be a marker for more aggressive disease that is less sensitive to local therapy. These patients may be less likely to benefit from non-operative management. Thus, especially careful patient selection for the watch-and-wait strategy is warranted for patients with stage III disease at this time. More studies are needed to clarify the appropriate role of non-operative management in patients with clinically node-positive disease at the time of diagnosis.

Similar to our study, Kalady et al. found an interval of ≥8 weeks from the end of CRT to surgery to be associated with improved pCR rates (34). In addition, a meta-analysis including 3584 patients showed that an interval >6–8 weeks is associated with a 6% increase in pCR rate (35). These findings provide guidance in determining the ideal time at which to evaluate patients for CRT response. As tumor killing with CRT is not immediate, an increased interval allows more patients who will eventually achieve a CR to be considered for non-operative management. Prospective studies of the watch-and-wait approach have evaluated patients for response at variable intervals, ranging from 4 to 10 weeks following CRT (7, 9, 10, 19–21). As evidence suggests that intervals ≥8 weeks may increase patients’ chances of rectal preservation, prospective studies are needed to determine the ideal time to evaluate for a CR.

Few studies have evaluated the import of RT treatment interruptions on achievement of a CR, one of the few modifiable factors that may affect outcomes. Our findings are consistent with the two other retrospective studies that have evaluated this treatment variable and have found no association (27, 36). Nevertheless, we found unplanned treatment breaks to be associated with greater risk of disease recurrence. Similarly, one of the above studies found duration of RT ≥40 days to be borderline associated with DFS (HR 4.45, 95% CI 0.99–20.1, *p* = 0.052) (27). In light of the available data, care should be taken to minimize RT interruptions for rectal cancer patients.

The dearth of consistent and accurate clinical predictors of CR across multiple studies reveals a need for more precise prognosticators. Our improving ability to classify tumors through genotyping and phenotyping holds great potential to enhance our ability to determine which patients are most likely to benefit from a watch-and-wait strategy (37). One study analyzed 47 primary tumors for 60 frequent mutations, and found that fewer patients with a mutation either pre- or post-CRT achieved a pCR compared to patients with wild-type tumors (3.3 vs. 23.5%, *p* = 0.05) (27). In addition, several retrospective studies have found associations between the NLR or circulating lymphocyte count and treatment response or long-term outcomes, suggesting a role of the immune system in determining sensitivity to CRT (38–41). In our study, we found no association between NLR and outcomes, but did find that increasing lymphocyte count predicted against distant failure. Although these peripheral markers are affected by a variety of inflammatory states and thus are not specific for CRT response, these findings hint at a role of the immune system in determining treatment response. In the future, tumor sampling with in-depth genotyping and immunophenotyping may improve risk stratification and reveal novel strategies to improve chemo- and radiosensitivity.

Our study has several limitations inherent to its retrospective nature. Our population was relatively small, and thus our study may not have the statistical power to detect differences in survival between CR and non-CR patients. Nevertheless, most studies assessing predictors of CR are small and retrospective in nature, and we believe that our findings will add to the understanding of CRT response. In addition, our median follow-up interval of 24.5 months is sub-optimal, limiting our ability to assess predictors of survival. This, in combination with the sample size, may explain why we did not identify CR as an independent predictor of outcomes. Nevertheless, both pCR and cCR has been associated with improved long-term outcomes, and thus we believe that identifying predictors of CR is clinically relevant despite the negative findings of our study (2, 19). Also, the missing concurrent chemotherapy data on 5.1% of patients was significant on regression analysis of locoregional recurrence and disease recurrence, limiting interpretation of these models. Finally, a subset of patients managed non-operatively was not managed per a formal watch-and-wait protocol. While it is possible that some of these patients did not in fact achieve a cCR, none of them have recurred at the time of preparation of this manuscript, and thus we believe it is reasonable to include them in the cCR group.

## Conclusion

In conclusion, we found higher CEA level at diagnosis, increased tumor size, increased distance from the anal verge, node-positive disease at diagnosis, and smaller interval from CRT to surgery to predict against CR to CRT. In our cohort, patients with a CR exhibited improved DMFS and DFS. These results are hypothesis generating and may aid in clinical decision making when considering patients for the watch-and-wait strategy. As this strategy is increasingly being adopted in clinical practice, larger prospective studies are needed to establish which clinicopathologic factors accurately predict CR.

## Author Contributions

DB designed the research; collected, analyzed, and interpreted the data; and wrote the manuscript. LS collected and interpreted the data and revised the manuscript. HM, NS, and PG contributed to research design, interpreted the data, and revised the manuscript. IH designed the research, analyzed and interpreted the data, and wrote the manuscript. KD directed and designed the research, analyzed and interpreted the data, and wrote the manuscript.

## Author Note

This research is accepted for poster presentation at the ASTRO 57th Annual Meeting on September 18–21, 2015.

## Conflict of Interest Statement

The authors declare that the research was conducted in the absence of any commercial or financial relationships that could be construed as a potential conflict of interest.
